# Central nervous system infiltration by HTLV-1-associated T-cell leukemia/lymphoma in an AIDS patient

**DOI:** 10.1590/0037-8682-0060-2020

**Published:** 2020-06-12

**Authors:** Luzia Beatriz Ribeiro Zago, Vanessa Afonso da Silva, Fernanda Bernadelli De Vito, Leonardo Rodrigues de Oliveira

**Affiliations:** 1 Universidade Federal do Triângulo Mineiro, Hospital de Clínicas, Serviço de Hematologia e Hemoterapia, Uberaba, MG, Brasil.; 2 Universidade Federal do Triângulo Mineiro, Hospital de Clínicas, Laboratório de Citometria de Fluxo, Uberaba, MG, Brasil.

A 59-year-old woman was admitted for progressive ataxia and decreased consciousness, which had commenced two months previously. The patient had *human immunodeficiency virus (*HIV)/*human*T-cell lymphotropic virus-1 (HTLV-1) co-infection for 10 years with regular use of antiretroviral therapy, resulting in satisfactory virological control (undetectable HIV load, CD4+ T lymphocyte count: 354 cells/mm³). Laboratory data revealed leukocytosis (142.3 × 10^9^/L - 78% lymphocytes, some with “flower cell” morphology [Figure 1]), hypercalcemia, elevated serum lactate dehydrogenase, and acute renal dysfunction without anemia or thrombocytopenia. Cranial computed tomography scans revealed calcification in basal ganglia. Cerebral spinal fluid (CSF) analysis revealed 30 cells/mm³ (86% atypical lymphocytes). Cytomegalovirus and *Toxoplasma gondii* IgM and IgG antibody screening were negative. No infectious agents were identified by CSF direct analysis and culture.

**FIGURE 1:**
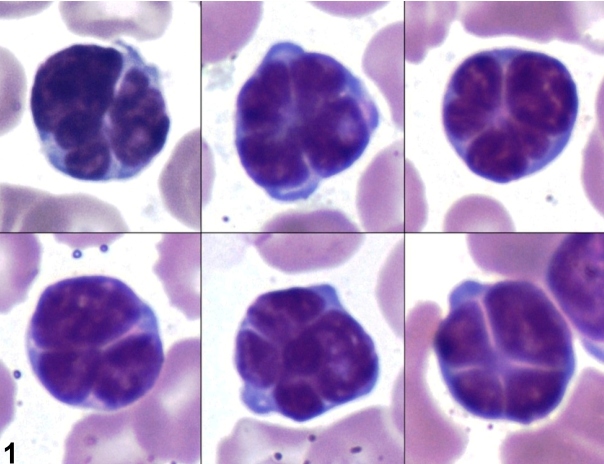
Atypical lymphocytes in peripheral blood showing classical “flower cell” morphology. Leishman stain, magnification × 1000.

Blood and CSF lymphocyte immunophenotyping by flow cytometry revealed positivity for CD3, CD4, CD5, CD25, and CD38 markers, and negativity for CD8 (Figure 2)^1^. A diagnosis of central nervous system infiltration (lymphomatous meningitis) by HTLV-1-associated adult T-cell leukemia/lymphoma (acute subtype) was considered^2^. No test for detecting clonal integration of the HTLV-1 pro-virus within tumor cells was conducted. Systemic and intrathecal chemotherapy were administrated. The patient died due to *Pseudomonas aeruginosa* infection 25 days later.

**FIGURE 2:**
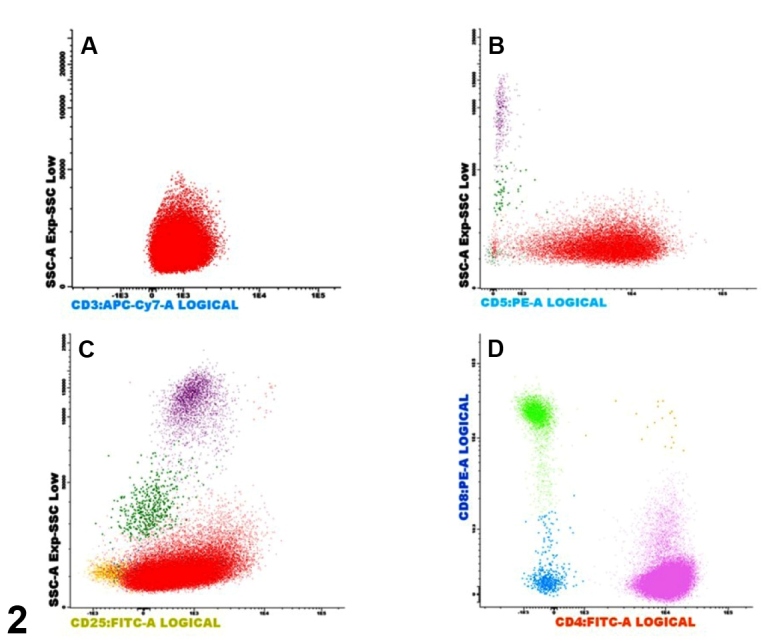
Blood lymphocyte immunophenotyping by flow cytometry. Lymphocytes (marked by red color in dot-plots) were CD3+ **(A)**, CD5+ **(B)**, and CD25+ (C). D: CD3+ T-lymphocytes with CD4+/CD8- phenotype (marked by pink color in dot-plots) in 97.3% of cells analyzed.

The spectrum of complications associated with HTLV-1 infection is broad, with predominant hematological and neurological manifestations^3^. The detection of lymphocytes with “flower cell” morphology may be useful for investigation of HTLV-1 infection. Guidelines for standardizing follow-up of patients with HTLV-1 infection should be considered for early detection of potential infection-related complications.

## References

[1] Bazarbachi A, Suarez F, Fields P, Hermine O (2011). How I treat adult T-cell leukemia/lymphoma. Blood.

[2] Shimoyama M (1991). Diagnostic criteria and classification of clinical subtypes of adult T-cell leukaemia-lymphoma. A report from the Lymphoma Study Group (1984-87). Br J Haematol.

[3] Araújo AQ, Leite AC, Lima MA, Silva MT (2009). HTLV-1 and neurological conditions: when to suspect and when to order a diagnostic test for HTLV-1 infection?. Arq Neuropsiquiatr.

